# The influence of tranexamic acid on intraoperative blood transfusion in short-segment PLIF and analysis of influencing factors of the transfusion: a retrospective case study

**DOI:** 10.3389/fphar.2026.1736382

**Published:** 2026-03-25

**Authors:** Shiying Luo, Xiaoya Zhu, Shenshen Hao, Fangshu Zhang, Shengli Dong, Shuai Liu, Hongke Li, Bowen Wang

**Affiliations:** 1 College of Chemistry and Chemical Engineering and Office of the Ombudsman, Pingdingshan University, Pingdingshan, Henan, China; 2 Interventional Operating Room, The Second People’s Hospital of Pingdingshan, Pingdingshan, Henan, China; 3 Department of Spine, Affiliated Hospital of Qingdao University, Qingdao, Shandong, China; 4 Department of Spine and Bone Oncology, General Hospital of Pingmei Shenma Medical Group, Pingdingshan, Henan, China; 5 Department of Orthopedics, Henan Provincial Third People’s Hospital, Zhengzhou, Henan, China; 6 Department of Hand Surgery, Norinco General Hospital, Xi’an, Shaanxi, China

**Keywords:** antifibrinolytic effect, intraoperative blood transfusion, posterior lumbar interbody fusion, protective factor, risk factor, tranexamic acid

## Abstract

**Background:**

Tranexamic acid (TXA), with the chemical name of trans-4-aminomethylcyclohexanecarboxylic acid, is a synthetic hemostatic drug with antifibrinolytic activity. However, there remains uncertainty regarding the effect of TXA in reducing intraoperative blood transfusion (IBT) during short-segment posterior lumbar interbody fusion (PLIF). Therefore, this study investigates the chemical and clinical factors related to IBT.

**Methods:**

A retrospective analysis was performed on the medical records of 402 patients who underwent short-segment PLIF between October 2020 and September 2024. Demographic indicators included age, gender, body mass index, history of hypertension, history of diabetes, and history of anticoagulation therapy. Laboratory test indicators included preoperative coagulation-related indicators and anemia-related indicators. Surgical-related indicators included disease type, surgical segment, operation time, intraoperative blood loss (IBL), and IBT. The drug-intervention factor was TXA. Patients who received intravenous TXA 15 min before surgery were classified as the observation group (97 cases), while those who did not were classified as the control group (305 cases). Univariate binary logistic regression analysis was used to explore the related influencing factors of IBT, and multivariate binary logistic regression analysis was further conducted to identify the independent influencing factors. A nomogram model was constructed to predict the probability of IBT.

**Results:**

Both IBL and IBT in the observation group were lower than those in the control group, and the differences were statistically significant (p < 0.05). Univariate regression analysis revealed that IBT was significantly correlated with TXA, disease type, surgical segment, red blood cell count, hemoglobin, platelet count, operation time, and IBL (p < 0.05). Multivariate regression analysis demonstrated a strong association between IBL and IBT (p < 0.001). Further analysis excluding IBL indicated that the independent influencing factors of IBT included TXA, disease type, surgical segment and operation time (p < 0.05). The nomogram model showed that TXA was a protective factor, while disease type, surgical segment and operation time were risk factors.

**Conclusion:**

In short-segment PLIF, TXA serves to reduce IBT by minimizing IBL. Moreover, it is an independent protective influencing factor for IBT. Conversely, the disease type, surgical segment, and operation time are independent risk influencing factors.

## 1 Background

Tranexamic acid (TXA) has a molecular formula of C_8_H_15_NO_2_, a molecular weight of 157.21 g/mol, and its chemical name is trans-4-aminomethylcyclohexanecarboxylic acid (Li and Chuan, 2024). Also referred to as hemostatic acid, it belongs to the category of artificially synthesized amino acid derivatives (Wang et al., 2024). In its molecular structure, the cyclohexane ring assumes a chair conformation, with the amino group and the carboxyl group in a trans configuration. This spatial arrangement endows TXA with the ability to specifically bind to the lysine-binding site of plasminogen. By this means, it blocks the transformation of plasminogen into plasmin, thereby suppressing the degradation of fibrin (Adigweme and Lee, 2017). When compared with natural lysine, the cyclohexane ring in the TXA molecule increases its hydrophobicity. This not only extends its half-life in the body but also renders it less susceptible to hepatic metabolism. Moreover, TXA is predominantly excreted in its native form via the kidneys (Andersson et al., 1978). Based on the aforementioned chemical properties, TXA exhibits a long-acting and stable hemostatic effect in clinical applications (Hong et al., 2022).

Short-segment posterior lumbar interbody fusion (PLIF) has been widely employed in in the treatment of lumbar degenerative diseases owing to its favorable therapeutic outcomes (Li et al., 2019; Mobbs et al., 2015). Nevertheless, intraoperative vascular injury and subsequent bleeding resulting from surgical maneuvers can trigger the activation of the body’s coagulation and fibrinolysis systems (Zhong et al., 2018). Upon vascular injury, coagulation factors, including factor VII, IX, and X, are activated via both the intrinsic and extrinsic pathways. These activated factors facilitate the conversion of fibrinogen (FIB) into fibrin, thereby forming blood clots as a means of hemostasis. Simultaneously, within the activated fibrinolysis system, plasminogen is transformed into plasmin under the influence of tissue-type plasminogen activator. Subsequently, plasmin degrades blood clots by hydrolyzing fibrin, which may give rise to bleed. In PLIF, significant intraoperative blood loss (IBL) episodes are not uncommon (Machado et al., 2017). Intraoperative blood transfusion (IBT) is frequently utilized to restore blood volume. However, this approach not only escalates healthcare costs but also subjects patients to potential risks such as hemolytic reactions and infections (Zhao et al., 2020; Park et al., 2024). Consequently, given the antifibrinolytic properties of TXA, the strategy of reducing IBL to minimize the requirement for IBT holds significant promise and feasibility in clinical practice.

However, there is a question as to whether a single intravenous injection of 1g of TXA 15 min before surgery yields such an effect. This was one of the typical dosing regimens recommended in the 2019 Chinese expert consensus (Zhou et al., 2019). This mode is characterized by safety, efficacy, simplicity, and feasibility, without interfering with intraoperative anesthesia (Yuan et al., 2021). Moreover, a multitude of factors are implicated in IBT ((Wang et al., 2021). It is of great significance for clinical practice to identify which are conservative factors and which are risks. Therefore, this study evaluates these issues.

## 2 Methods

### 2.1 Research design

This study was a single-center, retrospective case study. It was approved by the Ethics Committee of the General Hospital of Pingmei Shenma Medical Group, and the ethical approval code was 2021004. The time range for case collection was from October 2020 to September 2024. The inclusion criteria for this study were as follows. *a.* All patients were residents of the Asian region. *b*. The preoperative diagnosis was lumbar degenerative disease, including lumbar disc herniation (LDH), lumbar spinal stenosis (LSS), and lumbar spondylolisthesis (LS). *c*. The planned surgical segment had not undergone any previous surgeries. *d*. The surgical approach was PLIF. *e*. The surgical segment was either single-segment or double-segments. The exclusion criteria were as follows. *a*. There were hematological diseases before surgery. *b*. The patients had preoperative concurrent lumbar infectious diseases, fractures or tumors. *c*. There was a history of deep vein thrombosis (DVT) in the lower extremities before surgery. *d*. Intraoperative cerebrospinal fluid leakage occurred. *e*. The patients had rheumatic diseases.

### 2.2 Introduction to TXA and detection methods used in the research institute

The TXA (1g/100 mL) used in this study was produced by Chongqing Lummy Pharmaceutical Co., Ltd., China, with the batch number H20031101. Platelet count (PLT), hemoglobin (HB), red blood cell count (RBC), and hematocrit (HCT) were measured using an automated hematology analyzer manufactured by Shenzhen Mindray Bio-Medical Electronics Co., Ltd., China. Activated partial thromboplastin time (APTT) and prothrombin time (PT) were detected via the coagulation method, with instruments from Boston Instrument Laboratory Company, United States. Thrombin time (TT) was detected by the coagulation method, and FIB was measured by the Clauss method, with reagents from Wuhan Zhong Tai Biotech Co., Ltd., China.

### 2.3 Research methods

For patients who had been receiving aspirin anticoagulation therapy in the week before surgery, they were switched to low-molecular-weight heparin preparations or indobufen tablets (produced by Hangzhou Zhongmei Huadong Pharmaceutical Co., Ltd., China, with the batch number H20194067) and the medication was discontinued 1 day before surgery. For patients with hypertension, antihypertensive drugs were discontinued on the morning of the surgery day. For patients with diabetes, antidiabetic medications were discontinued on the evening before surgery. The application method of TXA was a single intravenous infusion of 1g of TXA 15 min before skin incision after general anesthesia. All patients underwent general anesthesia and standard PLIF procedures. Given the similarity of the surgical procedures, the key operative steps were outlined below (Hao et al., 2023a). A longitudinal incision was centered over the diseased lumbar spinal segment. Initially, the skin and subcutaneous tissues were dissected layer by layer to the level of the spinous process, followed by subperiosteal dissection of bilateral paravertebral muscles to expose relevant osseous and articular structures, including the spinous process, lamina, and facet joints. Subsequent procedural steps included pedicle screws fixation, decompressive laminectomy, discectomy and interbody fusion. Finally, meticulous hemostasis was achieved, the incision was closed in anatomical layers, and negative-pressure drainage tubes were placed. The diagnosis of postoperative DVT was strictly based on lower extremity vascular Doppler ultrasound findings, rather than mere inference from clinical symptoms alone. Doppler ultrasound examinations were indicated when patients exhibited clinical manifestations suggestive of DVT, including: sudden onset of pain in unilateral or bilateral lower extremities, alterations in skin temperature, and asymmetry in bilateral limb circumference.

The criteria for IBT were referenced as follows (Zhao et al., 2020). *a.* No blood transfusion was indicated when the intraoperative HB level exceeds 100 g/L. *b*. Blood transfusion was required if the HB level was less than 70 g/L. *c*. For patients with HB levels ranging from 70 g/L to 100 g/L, the necessity of blood transfusion shall be determined by a joint assessment of the operating surgeons and anesthesiologist. The specific clinical triggers for the “joint assessment” were outlined as follows. Firstly, persistent oozing or active bleeding from the surgical incision without a discernible trend of reduction, as evaluated and confirmed by the surgeon. Secondly, hemodynamic instability, characterized by persistent hypotension with suboptimal response to volume resuscitation, as assessed and confirmed by the anesthesiologist. Thirdly, abnormally elevated heart rate presenting as hypovolemia-related tachycardia, as evaluated and confirmed by the anesthesiologist.

### 2.4 Research indicators

Demographic indicators included age, gender, body mass index (BMI), history of hypertension, history of diabetes, and history of anticoagulation therapy. Coagulation-related indicators included preoperative APTT, PT, TT, FIB, and PLT. Anemia-related indicators included HB, RBC, and HCT. Surgical-related indicators included disease type, surgical segment, operation time, IBL, IBT, and postoperative lower extremity DVT. Drug-intervention factor was whether the patient received TXA before surgery.

### 2.5 Statistical methods

The collected data were analyzed using R statistical software (version 4.0.3). IBL in the groups was compared by the independent sample t-test. The number of IBT was compared via the chi-square test. Univariate binary logistic regression analysis was conducted through the glm function. Specifically, each variable was entered into the model individually. When the independent variable was a categorical variable, the group with the minimum value was set as the reference group. When the independent variable was a continuous variable, it was directly incorporated into the binary logistic regression model. The receiver operating characteristic curve was utilized, and the value corresponding to the maximum Youden index was chosen as the cut-off value. The continuous variable was then transformed into a binary variable, with the suffix “To2c” added to the variable name, followed by binary logistic regression analysis. Variables that showed significant significance in the univariate analysis were used as independent variables for multivariate binary logistic regression analysis using the glm function. A nomogram model was constructed to predict the probability of IBT. A p-value <0.05 was considered statistically significant.

## 3 Results

### 3.1 General information and blood loss

Based on the aforementioned inclusion and exclusion criteria, a total of 402 eligible cases were enrolled. Among the patients in this study, all surgeries were successfully performed, without postoperative DVT observed. 55 cases received IBT, while 347 cases did not. The overall IBT rate was 13.7%. The demographic indicators, coagulation-related indicators, anemia-related indicators, surgical-related indicators, and drug-intervention factor were detailed in Tables 1, 2.

**TABLE 1:** The descriptive statistics of general data (n = 402).

Variable	Mean ± standard deviation
Age, y	60.770 ± 10.285
BMI, kg/m^2^	25.208 ± 3.315
PT, s	11.115 ± 0.767
APTT, s	31.263 ± 2.928
FIB, g/L	2.958 ± 0.511
TT, s	14.708 ± 1.122
RBC, 10^12^/L	4.424 ± 0.433
HB, g/L	136.910 ± 14.240
HCT, L/L	0.413 ± 0.063
PLT, 10^9^/L	220.640 ± 66.269
Operation time, min	174.520 ± 41.863
IBL, mL	364.550 ± 188.147

BMI, body mass index; PT, prothrombin time; APTT, activated partial thromboplastin time; FIB, fibrinogen; TT, thrombin time; RBC, red blood cell count; HB, hemoglobin; HCT, hematocrit; PLT, platelet count; IBL, intraoperative blood loss.

**TABLE 2:** The frequency description of general data (n = 402).

Variable		Frequency	Percentage (%)
Gender, n	Male	210	52.2
	Female	192	47.8
TXA, n	Yes	97	24.1
	No	305	75.9
History of anticoagulation therapy, n	Yes	106	26.4
	No	296	73.6
History of hypertension, n	Yes	122	30.3
	No	280	69.7
History of diabetes, n	Yes	60	14.9
	No	342	85.1
Disease type, n	LS	93	23.1
	LDH	100	24.9
	LSS	209	52.0
Surgical segment, n	One	222	55.2
	Two	180	44.8
IBT, n	Yes	55	13.7
	No	347	86.3

TXA, tranexamic acid; IBT, intraoperative blood transfusion.

### 3.2 Comparison between the groups

There were 97 cases in the observation group and 305 cases in the control group. The IBL and IBT in the observation group was less than those in the control group, and the differences were statistically significant (p < 0.05). The comparison results of the two groups were shown in Table 3.

**TABLE 3:** The comparison of IBL and IBT between the two groups.

Observation group (n = 97)		Control group (n = 305)	t/χ^2^	P
IBL, mL	323.71 ± 152.085	377.54 ± 196.695	−2.470	0.014^a^
IBT, n	​	​	4.525	0.033^b^
Yes	7	48	​	​
No	90	257	​	​

IBL, intraoperative blood loss; IBT, intraoperative blood transfusion; a, denotes the result of the independent sample t-test; b, denotes the result of the chi-square test.

### 3.3 Univariate binary logistic regression analysis of IBT

Univariate binary logistic regression analysis indicated that TXA, disease type, surgical segment, RBC, HB, PLT, operation time, and IBL were significantly associated with IBT (p < 0.05). In contrast, age, gender, BMI, history of hypertension, history of diabetes, and history of anticoagulation therapy, APTT, and HCT were not significantly associated with IBT (p > 0.05). The results were presented in Table 4.

**TABLE 4:** The univariate binary logistic regression analysis of IBT in short-segment PLIF.

Variable	B	SE	z	p	OR [95%CI]
Gender, n (Ref:0) (1 = male, 0 = female)	−0.23	0.291	−0.792	0.428	0.79 [0.45,1.4]
Age, y	0.010	0.014	0.718	0.473	1.01 [0.98,1.04]
Age To2c	​	​	​	​	​
≤60.5	Ref.	​	​	​	​
>60.5	0.108	0.296	0.364	0.716	1.11 [0.62,1.99]
TXA, n (Ref:0) (1 = yes, 0 = no)	−0.876	0.423	−2.072	0.038	0.42 [0.18,0.95]
History of anticoagulation therapy, n (Ref:0) (1 = yes, 0 = no)	0.359	0.313	1.148	0.251	1.43 [0.78,2.64]
History of hypertension, n (Ref:0) (1 = yes, 0 = no)	−0.28	0.331	−0.848	0.397	0.76 [0.4,1.44]
History of diabetes, n (Ref:0) (1 = yes, 0 = no)	−0.212	0.431	−0.492	0.623	0.81 [0.35,1.88]
BMI, kg/m^2^	−0.017	0.044	−0.376	0.707	0.98 [0.9,1.07]
BMI To2c	​	​	​	​	​
≤25.490	Ref.	​	​	​	​
>25.490	0.307	0.291	1.055	0.291	1.36 [0.77,2.4]
Disease type, n (Ref:0) (0 = LS,1 = LDH,2 = LSS)	​	​	​	​	​
0	Ref.	​	​	​	​
1	−1.18	0.503	−2.348	0.019	0.31 [0.11,0.82]
2	−0.103	0.334	−0.308	0.758	0.9 [0.47,1.74]
Surgical segment, n (Ref:1) (1 = one,2 = two)	1.703	0.344	4.945	<0.001	5.49 [2.8,10.79]
PT, s	−0.07	0.192	−0.364	0.716	0.93 [0.64,1.36]
PT To2c	​	​	​	​	​
≤11.15	Ref.	​	​	​	​
>11.15	−0.296	0.295	−1.001	0.317	0.74 [0.42,1.33]
APTT, s	0.017	0.049	0.349	0.727	1.02 [0.92,1.12]
APTT To2c	​	​	​	​	​
≤33.65	Ref.	​	​	​	​
>33.65	0.584	0.334	1.747	0.081	1.79 [0.93,3.45]
FIB, g/L	−0.167	0.291	−0.575	0.565	0.85 [0.48,1.5]
FIB To2c	​	​	​	​	​
≤2.765	Ref.	​	​	​	​
>2.765	0.272	0.304	0.895	0.371	1.31 [0.72,2.38]
TT, s	0.086	0.128	0.673	0.501	1.09 [0.85,1.4]
TT To2c	​	​	​	​	​
≤16.925	Ref.	​	​	​	​
>16.925	−14.765	641.305	−0.023	0.982	0 [0,Inf]
RBC, 10^12^/L	−0.266	0.338	−0.788	0.43	0.77 [0.4,1.49]
RBC To2c	​	​	​	​	​
≤4.585	Ref.	​	​	​	​
>4.585	−0.697	0.336	−2.074	0.038	0.5 [0.26,0.96]
HB, g/L	−0.014	0.010	−1.333	0.183	0.99 [0.97,1.01]
HB To2c	​	​	​	​	​
≤144.5	Ref.	​	​	​	​
>144.5	−0.815	0.368	−2.217	0.027	0.44 [0.22,0.91]
HCT, L/L	−4.713	3.573	−1.319	0.187	0.01 [0,9.88]
HCT To2c	​	​	​	​	​
≤0.425	Ref.	​	​	​	​
>0.425	−0.587	0.329	−1.785	0.074	0.56 [0.29,1.06]
PLT	−0.002	0.002	−1.015	0.31	1 [0.99,1]
PLT, 10^9^/L To2c	​	​	​	​	​
≤177.5	Ref.	​	​	​	​
>177.5	−0.854	0.299	−2.857	0.004	0.43 [0.24,0.76]
Operation time, min	0.026	0.004	6.525	<0.001	1.03 [1.02,1.03]
Operation time To2c	​	​	​	​	​
≤197	Ref.	​	​	​	​
>197	1.971	0.311	6.334	<0.001	7.18 [3.9,13.21]
IBL, mL	0.011	0.001	8.382	<0.001	1.01 [1.01,1.01]
IBL To2c	​	​	​	​	​
≤575	Ref.	​	​	​	​
>575	3.818	0.390	9.784	<0.001	45.5 [21.18,97.75]

TXA, tranexamic acid; BMI, body mass index; LS, lumbar spondylolisthesis; LDH, lumbar disc herniation; LSS, lumbar spinal stenosis; PT, prothrombin time; APTT, activated partial thromboplastin time; FIB, fibrinogen; TT, thrombin time; RBC, red blood cell count; HB, hemoglobin; HCT, hematocrit; PLT, platelet count; IBL, intraoperative blood loss; IBT, intraoperative blood transfusion; B, unstandardized beta; SE, standard error; OR, odds ratio; CI, confidence Interval; PLIF, posterior lumbar interbody fusion.

### 3.4 Multivariate binary logistic regression analysis of IBT

Since IBL determined whether IBT was required during surgery, there was a strong correlation between the two. When IBL was incorporated into the analysis, regression analysis revealed that it was an independent influencing factor for IBT (p < 0.001). Under this circumstance, other potentially factors may be masked. The details were presented in Table 5.

**TABLE 5:** The multivariate binary logistic regression analysis of IBT in short-segment PLIF.

Variable	B	SE	z	p	OR [95%CI]
Constant	−4.24	2.541	−1.669	0.095	​
TXA, n (Ref:0) (1 = yes, 0 = no)	−0.89	0.569	−1.565	0.118	0.41 [0.13,1.25]
Disease type, n (Ref:0) (0 = LS,1 = LDH,2 = LSS)	​	​	​	​	​
0	Ref.	​	​	​	​
1	−1.015	0.764	−1.329	0.184	0.36 [0.08,1.62]
2	0.384	0.515	0.746	0.455	1.47 [0.54,4.03]
Surgical segment, n (Ref:1) (1 = one,2 = two)	0.769	0.481	1.598	0.11	2.16 [0.84,5.54]
RBC, 10^12^/L	−1.049	0.899	−1.166	0.244	0.35 [0.06,2.04]
HB, g/L	−0.001	0.025	−0.026	0.98	1 [0.95,1.05]
PLT, 10^9^/L	−0.001	0.003	−0.306	0.76	1 [0.99,1.01]
Operation time, min	0.007	0.006	1.223	0.221	1.01 [1,1.02]
IBL	0.01	0.002	6.843	<0.001	1.01 [1.01,1.01]

TXA, tranexamic acid; LS, lumbar spondylolisthesis; LDH, lumbar disc herniation; LSS, lumbar spinal stenosis; RBC, red blood cell count; HB, hemoglobin; PLT, platelet count; IBL, intraoperative blood loss; IBT, intraoperative blood transfusion; B, unstandardized beta; SE, standard error; OR, odds ratio; CI, confidence Interval; PLIF, posterior lumbar interbody fusion.

Therefore, it was necessary to exclude IBL and then performed a multivariate binary logistic regression analysis. The analysis after excluding IBL demonstrated that TXA, disease type, surgical segments, and operation time were independent influencing factors for IBT (p < 0.05). The details were presented in Table 6.

**TABLE 6:** The multivariate binary logistic regression analysis of IBT after excluding IBL in short-segment PLIF.

Variable	B	SE	z	p	OR [95%CI]
Constant	−5.17	2.049	−2.523	0.012	​
TXA, n (Ref:0) (1 = yes, 0 = no)	−1.28	0.474	−2.700	0.007	0.28 [0.11,0.7]
Disease type, n (Ref:0) (0 = LS,1 = LDH,2 = LSS)	​	​	​	​	​
0	Ref.	​	​	​	​
1	−1.299	0.570	−2.277	0.023	0.27 [0.09,0.83]
2	−0.282	0.410	−0.687	0.492	0.75 [0.34,1.69]
Surgical segment, n (Ref:1) (1 = one,2 = two)	1.138	0.406	2.804	0.005	3.12 [1.41,6.91]
RBC, 10^12^/L	0.166	0.649	0.256	0.798	1.18 [0.33,4.21]
HB, g/L	−0.018	0.019	−0.933	0.351	0.98 [0.95,1.02]
PLT, 10^9^/L	−0.002	0.002	−0.939	0.348	1 [0.99,1]
Operation time, min	0.023	0.004	5.261	<0.001	1.02 [1.01,1.03]

TXA, tranexamic acid; LS, lumbar spondylolisthesis; LDH, lumbar disc herniation; LSS, lumbar spinal stenosis; RBC, red blood cell count; HB, hemoglobin; PLT, platelet count; IBL, intraoperative blood loss; IBT, intraoperative blood transfusion; B, unstandardized beta; SE, standard error; OR, odds ratio; CI, confidence Interval; PLIF, posterior lumbar interbody fusion.

### 3.5 Nomogram model of independent predictors for IBT

**FIGURE 1:**
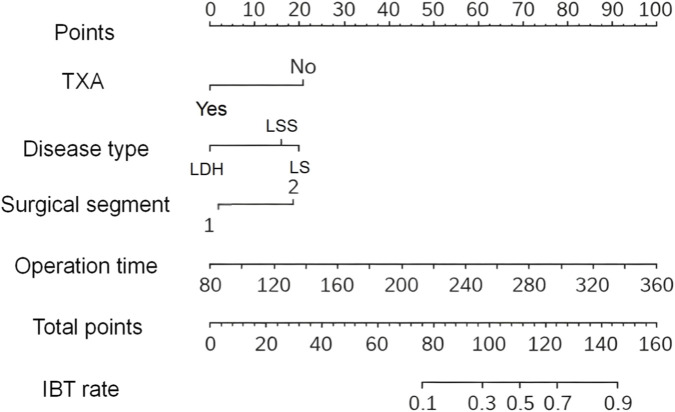
The nomogram model of IBT risk in short-segment PLIF. TXA: The decision of whether to receive TXA significantly impacted the risk of IBT. Patients who were administered TXA had a lower likelihood of requiring IBT, whereas those without TXA usage faced a higher risk of IBT. Disease type: Diseases such as LSS and LS are associated with a higher risk of IBT. This finding suggested that the severity or type of the disease directly influences a patient’s need for IBT. Surgical segment: The surgical segment affected the overall score, thereby influencing the probability of IBT. Patients undergoing surgery on two segments required more blood support compared to those on 1 segment. Consequently, the probability of IBT for patients in two segments was higher. Operation time: As the operation time prolonged, the overall score gradually increased, subsequently elevating the risk of IBT. When the surgical procedure took longer, patients were more likely to need additional blood support. TXA, tranexamic acid; IBL, intraoperative blood loss; IBT, intraoperative blood transfusion; PLIF, posterior lumbar interbody fusion.

The nomogram model could predict the probability of IBT by converting various variables into scores and summing them up. The analysis indicated that TXA, disease type, surgical segment, and operation time all had a significant impact on the risk of IBT. Specifically, the higher the total score of the model, the greater the probability of IBT. Details were presented in Figure 1.

### 3.6 Curve plot of independent predictors of IBT

**FIGURE 2:**
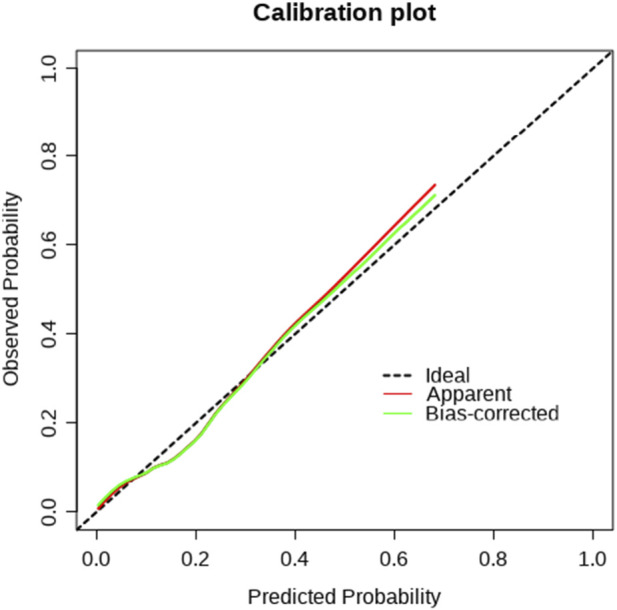
The calibration curve plot of IBT risk in short-segment PLIF. Ideal Curve: This dashed line represented the perfect calibration model, where the observed probabilities precisely coincided with the predicted probabilities. If the model’s predictions matched the actual observations, this line would overlap with the ideal curve. Apparent Curve: This red curve depicted the original prediction results of the model, which were the predicted probabilities calculated directly from the data. If this curve deviated from the ideal curve, it indicated that there is a certain degree of deviation in the model’s predictions. Bias-corrected Curve: This green curve represented the prediction results after bias correction. The bias of the original model was rectified through calibration methods, enabling the predictions to be closer to the actual observations. IBT, intraoperative blood transfusion; PLIF, posterior lumbar interbody fusion.

Calibration curve plots were employed to assess the accuracy of the predicted probabilities of the model. If the calibrated curve (green) closely approximated the ideal curve (black dashed line), it suggested that the calibrated model demonstrates good calibration performance, and the prediction outcomes are relatively accurate. There were depicted in Figure 2.

### 3.7 Threshold graph of independent predictors of IBT

**FIGURE 3:**
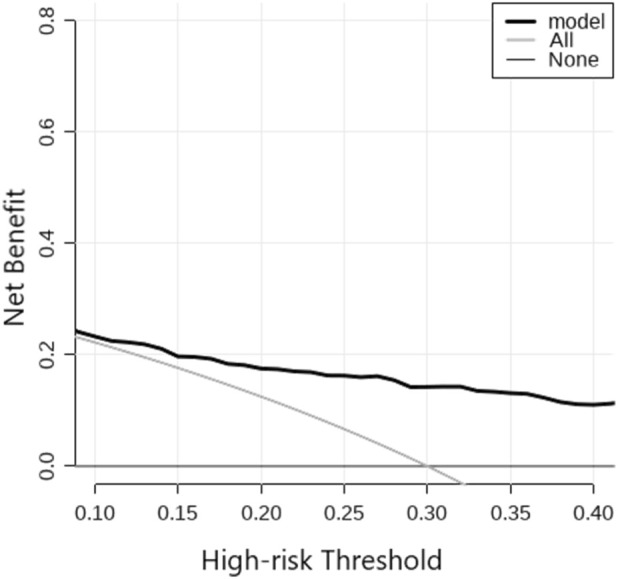
The threshold graph of IBT risk in short-segment PLIF. The black line (model): it represented the net benefit of the actually-utilized model. As the high-risk threshold increased, the net benefit gradually declined, suggesting that as the model’s discrimination ability for high-risk cases improves, its performance also undergoes changes. The gray line (All): it represented the scenario where all patients were regarded as high-risk cases. It presented a relatively flat net-benefit curve, reflecting the outcome when all individuals were considered to require treatment. The light-gray line (None): it represented the scenario where all patients were treated as low-risk cases, namely, the net benefit in the absence of any treatment intervention. IBT, intraoperative blood transfusion; PLIF, posterior lumbar interbody fusion.

The threshold graph illustrated the relationship between the high-risk threshold and the net benefit. It was commonly utilized to assess the predictive performance and risk stratification of the model. When the high-risk threshold was relatively low, the net benefit of the model (represented by the black line) was high, suggesting that the model identifies a greater number of high-risk individuals at this point. As the high-risk threshold increased, the net benefit of the model gradually declined. However, it consistently outperformed the scenarios where all individuals were considered as low-risk (the light-gray line) or high-risk (the gray line). The threshold graph revealed that the model could yield better clinical benefits when the threshold probability ranged from 0.01 to 0.97. The specific details were presented in Figure 3.

## 4 Discussion

There are two main reasons contributing to the significant IBL in short-segment PLIF. One is attributed to the characteristics of the surgery itself. The surgical procedure involves a relatively large surgical field, long operation duration, and persistent bleeding during the operation. Intraoperative vascular injury can synchronously activate the coagulation system and fibrinolysis system (Zhong et al., 2018). The other is the augmented activity of the fibrinolysis system during the surgery. According to a previous study, in the context of surgical bleeding, bleeding resulting from hyperfibrinolysis constitutes 60% of the total blood loss (Liu et al., 2011). Elevated blood loss can obscure the surgical field, increase procedural complexity, and potentially prolong operative duration—further exacerbating IBL and the requirement for IBT.

TXA diminishes bleeding by suppressing the fibrinolytic system (Dong et al., 2025). The underlying mechanism involves it mimicking the structure of lysine to bind with plasminogen (Duan et al., 2025). This binding inhibits the activation of plasmin and its interaction with fibrin, thereby preventing the dissolution of blood clots and fulfilling a hemostatic function (Kang et al., 2019). Consistent with previous studies, TXA serves as a hemostatic agent in PLIF (Hao et al., 2023b; Dong et al., 2025; Hao et al., 2023a). Meanwhile, it does not elevate the risk of postoperative DVT, making it a safe and reliable therapeutic option (Luan et al., 2023; Kim et al., 2017). Reduced bleeding contributes to a decrease in procedural complexity and surgical difficulty, thereby shortening operative duration and reducing the requirement for IBT. The findings of this study confirm that TXA can effectively reduce IBL and the demand for IBT. Furthermore, through multivariate regression analysis and the nomogram model, it is established that TXA is an independent influencing factor for IBT, and more specifically, a protective factor.

Typically, numerous factors can influence IBT (Wang et al., 2021). Among these, IBL often serves as a crucial determinant for surgeons when deciding whether to administer IBT (Lang et al., 2023). There exists a substantial correlation between the two variables. The preliminary multiple regression analysis conducted in this study also supports this association. Simultaneously, it suggests that IBL may exert a potent confounding effect, potentially obscuring the impact of other contributing factors. Therefore, it is imperative to exclude the influence of IBL prior to subsequent analyses. The results of the second multivariate regression analysis revealed that TXA, surgical segment, operation time, and disease type are independent factors influencing IBT. Through the nomogram model, it was further corroborated that TXA acts as a protective factor, whereas the surgical segment, operation time, and disease type are identified as risk factors. Generally speaking, it is intuitive that as the disease progresses from LDH to LSS and then to LS, the complexity of the disease and the intricacy of the surgical procedure gradually increase (Zhao et al., 2020). Likewise, an increase in the number of surgical segments and an elongation of the operative duration both contribute to augmented IBL (Aoude et al., 2016; Qiu et al., 2020). According to our data, these factors collectively lead to an elevated demand for TBT.

There exists such a phenomenon that patients who need to maintain anticoagulant therapy prior to surgery are commonly encountered. The use of oral anticoagulant medications, such as aspirin tablets, elevates the risk of IBL (Cheng et al., 2017). Therefore, alternative medications are necessary to sustain anticoagulant therapy before surgery. A frequently employed method is the subcutaneous injection of low-molecular-weight heparin preparations (Shiu et al., 2018). Moreover, for patients who are reluctant to adopt this approach, oral indobufen tablets represent an option. Indobufen is an oral medication that exhibits anticoagulant effects comparable to those of aspirin at equivalent dosages (Patrono et al., 2008). The preoperative administration of anticoagulant drugs could disrupt the equilibrium between coagulation and anticoagulation within the body. Thus, in theory, it may exacerbate IBL, thereby increasing the requirement for IBT. However, there is a paucity of research regarding whether the preoperative use of anticoagulant drugs augments IBT in short-segment PLIF. The results of this study indicate that the preoperative application of anticoagulant drugs is not an independent determinant influencing IBT. Furthermore, in our prior research, it was discovered that the preoperative use of anticoagulant drugs did not impinge on the hemostatic efficacy of intravenous 1g of TXA in patients undergoing double-segment PLIF (Hao et al., 2024). These findings may offer a certain degree of reference for the preoperative management of such patients. In addition, several factors—including advanced age, preoperative HB levels, and gender—have been reported in prior literature as being significantly associated with IBT (Qiu et al., 2020; Zhu and Wang, 2022; Chen et al., 2019). However, analogous findings were not observed in the present study. A potential explanation for this discrepancy lies in the imbalance of patient baseline characteristics across different studies.

## 5 Limitations

However, this study does have several limitations. Firstly, as a single-center retrospective study, the sample size of included cases is relatively small. A limited sample may not comprehensively represent the entire population, potentially restricting the generalizability of the research findings. Secondly, the study is subject to uncontrollable potential biases, including patient selection bias, lack of randomization, measurement errors, and potential confounding factors. Thirdly, IBL is a crucial determinant for IBT. When incorporated into the multivariate regression analysis framework, it has the potential to obscure the identification of other associated influencing factors. Consequently, it becomes essential to exclude IBL from the analysis and then re-perform the multivariate regression analysis. This approach, although aiming to isolate the effects of other factors, could potentially undermine the rigor of the multivariate regression analysis. Fourthly, the present study was primarily focused on IBL and IBT decision-making, with postoperative total blood loss and postoperative blood transfusion not incorporated into the analysis. This represents an unavoidable limitation of the current research. In future studies, these aspects will be subjected to further investigation. Therefore, it is highly necessary to conduct multi-center, prospective or randomized controlled trials in the future to improve the generalizability and robustness of research findings, thereby providing more reliable evidence for clinical practice.

## 6 Conclusion

In this study, we observed that TXA reduces IBT in short-segment PLIF by decreasing IBL. Concurrently, a comprehensive analysis and identification of the relevant factors influencing IBT were conducted. Specifically, TXA was determined to be a protective factor among the independent influencing factors. Conversely, disease type, surgical segment, and surgical duration were identified as risk factors. These findings contribute to a deeper understanding of the mechanisms underlying IBT during PLIF and may provide valuable guidance for future clinical practice and research.

## Data Availability

The original contributions presented in the study are included in the article/supplementary material, further inquiries can be directed to the corresponding author.
